# Mechanomedicine for Addressing Skeletal Muscle Insulin Resistance

**DOI:** 10.1210/endrev/bnaf012

**Published:** 2025-04-24

**Authors:** Lu Wang, Le Chang, Yufei Ma, Yuanbo Jia, Bin Gao, Wei Cui, Feng Xu

**Affiliations:** Department of Geriatric Endocrinology & Metabolism (Second Department of Geriatric Internal Medicine), The First Affiliated Hospital of Xi'an Jiaotong University, Xi'an 710061, P.R. China; The Key Laboratory of Biomedical Information Engineering of Ministry of Education, School of Life Science and Technology, Xi’an Jiaotong University, Xi’an 710049, P.R. China; Bioinspired Engineering & Biomechanics Center (BEBC), Xi'an Jiaotong University, Xi’an 710049, P.R. China; Bioinspired Engineering & Biomechanics Center (BEBC), Xi'an Jiaotong University, Xi’an 710049, P.R. China; Shaanxi Provincial Key Laboratory of Infection and Immune Diseases, Shaanxi Provincial People's Hospital, Xi’an 710068, P.R. China; The Key Laboratory of Biomedical Information Engineering of Ministry of Education, School of Life Science and Technology, Xi’an Jiaotong University, Xi’an 710049, P.R. China; Bioinspired Engineering & Biomechanics Center (BEBC), Xi'an Jiaotong University, Xi’an 710049, P.R. China; Bioinspired Engineering & Biomechanics Center (BEBC), Xi'an Jiaotong University, Xi’an 710049, P.R. China; Department of Transplant surgery, The Second Affiliate Hospital of Xi’an Jiaotong University, Xi’an 710004, P.R. China; Department of Endocrinology, The Second Affiliated Hospital of Air Force Medical University, Xi’an 710038, P.R. China; Department of Geriatric Endocrinology & Metabolism (Second Department of Geriatric Internal Medicine), The First Affiliated Hospital of Xi'an Jiaotong University, Xi'an 710061, P.R. China; International Obesity and Metabolic Disease Research Center (IOMDRC), Xi’an Jiaotong University, Xi’an 710061, P.R. China; The Key Laboratory of Biomedical Information Engineering of Ministry of Education, School of Life Science and Technology, Xi’an Jiaotong University, Xi’an 710049, P.R. China; Bioinspired Engineering & Biomechanics Center (BEBC), Xi'an Jiaotong University, Xi’an 710049, P.R. China

**Keywords:** skeletal muscle, insulin resistance, stiffness, mechanosensitive proteins, biomechanics, mechanobiology

## Abstract

Skeletal muscle insulin resistance (IR) is a critical deficiency in IR pathophysiology that substantially affects overall metabolic health. Skeletal muscle is mechanically sensitive since its structure and function are significantly influenced by factors such as mechanical stretching and tissue stiffness. These mechanical stimuli can cause adaptive changes that enhance muscle performance and resilience. In this review, we discuss the current state of skeletal muscle IR research from the perspective of mechanomedicine. We also systematically and comprehensively present the evolution of mechanomedicine in addressing skeletal muscle IR by various disciplines, including biomechanics, mechanobiology, mechanodiagnosis, and mechanotherapy. The goal of the review is to provide important theoretical insights and practical methods for elucidating the pathogenesis of IR and to advance diagnostic and therapeutic approaches informed by mechanomedicine.

## Graphical Abstract

**Graphical Abstract:**
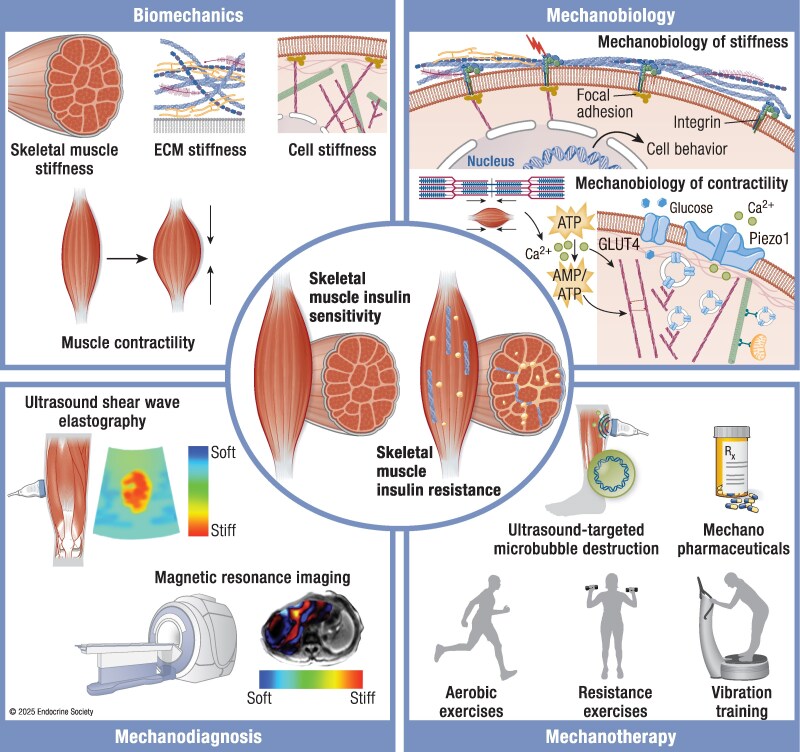

Essential points

- Skeletal muscle is pivotal in maintaining systemic glucose homeostasis and constitutes the primary site of whole-body insulin resistance (IR), positioning it as a critical target for IR intervention
- As a mechanosensitive tissue, skeletal muscle offers novel mechanomedical opportunities for advancing research on IR
- IR mechanically increases stiffness in skeletal muscle cells and the extracellular matrix (ECM), impairing contractility, disrupting force transmission, and directly influencing glucose metabolism (eg, glucose uptake), thereby linking biomechanics to metabolic dysfunction
- Mechanotransduction in skeletal muscle involves ECM stiffness sensing via transmembrane complexes (eg, integrins), which relay mechanical signals through the cytoskeleton to the nucleus, modulating transcription factors (eg, YAP/TAZ) to regulate metabolism
- Shear wave elastography (integrated with ultrasound/magnetic resonance imaging) has emerged as a clinically noninvasive tool for diagnosing IR-associated skeletal muscle stiffness
- Mechanotherapy strategies, including diverse exercise modalities (eg, active exercise, passive stretching), pharmacological agents targeting mechanosensitive proteins, and exercise-mimicking drugs, have proven effective in improving skeletal muscle IR through various mechanisms

Insulin resistance (IR) is characterized by elevated insulin levels, which is a compensatory mechanism of the human body to maintain normoglycemia (1), and is closely linked to the etiology of other metabolic disorders, such as type 2 diabetes mellitus (T2DM) (2), nonalcoholic fatty liver disease (NAFLD) (3, 4), and sarcopenia (5). Therefore, it is essential that diagnosis and treatment of IR be timely and effective. Skeletal muscle accounts for approximately 80% of insulin-mediated glucose uptake, so it has a critical role in maintaining glucose homeostasis, and it is the primary contributor to whole-body IR (6, 7). Therefore, skeletal muscle potentially can greatly improve the management of IR and its associated metabolic disorders by addressing skeletal muscle dysfunction (6-8).

Skeletal muscle IR research has primarily focused on biochemical pathways, such as those involving insulin signaling, inflammatory responses, and disruptions in lipid metabolism (7, 9). However, skeletal muscle is a typical mechanosensitive tissue, which has led to new areas of IR research (10-16). Mechanomedicine is an innovative approach rooted in biomechanics and mechanobiology that encompasses both diagnostic and therapeutic strategies to advance medical science (17-19). From a biomechanics perspective, quantitative analysis of the mechanical properties of organisms across multiple scales has revealed that skeletal muscle undergoes stiffness changes and active contraction and force transmission reduction due to IR (13, 20-27). From a mechanobiological perspective, which focuses on how cells generate, sense, and respond to mechanical cues in their microenvironment (18, 28, 29), alterations in the stiffness of organisms across multiple scales can affect mechanosensitive receptors, such as integrins, on skeletal muscle cells. These receptors transmit mechanical signals from the extracellular matrix (ECM) to cells, influencing glucose metabolism and the nuclear morphology and transcriptional function (12). The decreased force generation and transmission of active contraction also are associated with glucose uptake and utilization. These factors highlight the complex interplay between mechanical cues and muscle metabolism in IR in skeletal muscle (23, 25, 26).

Mechanomedicine also can promote innovative approaches to mechanodiagnosis and mechanotherapy. Mechanodiagnosis assesses health by detecting differences in the biomechanical and mechanobiological characteristics of the organism (17). Mechanotherapy involves interventions that reduce or reverse injury to damaged tissues or promote the homeostasis of healthy tissues through mechanical means at the molecular, cellular, or tissue level (30, 31). Novel methods based on these approaches include ultrasound and magnetic resonance imaging (MRI) for assessing the mechanical properties of skeletal muscle (eg, stiffness), tailored exercise programs that optimize muscle stretching and contraction, and innovative therapeutic methods targeting biomechanical properties and mechanobiological pathways (32-34). Such interventions are considerably different from conventional medical approaches, providing new approaches for effectively diagnosing and treating skeletal muscle IR.

This review discusses the biomechanical characteristics of skeletal muscle influenced by IR and presents the mechanobiological mechanisms of skeletal muscle IR. We also present the existing approaches to mechanical diagnosis and treatment and then suggest potential future research directions that could lead to advancements in the field. The mechanomedical pathways are elucidated to encourage promising new strategies for enhancing the understanding, diagnosis, and treatment of skeletal muscle IR.

## Evolution of the Understanding of Skeletal Muscle Insulin Resistance From the Perspective of Mechanomedicine

**Figure 1.**
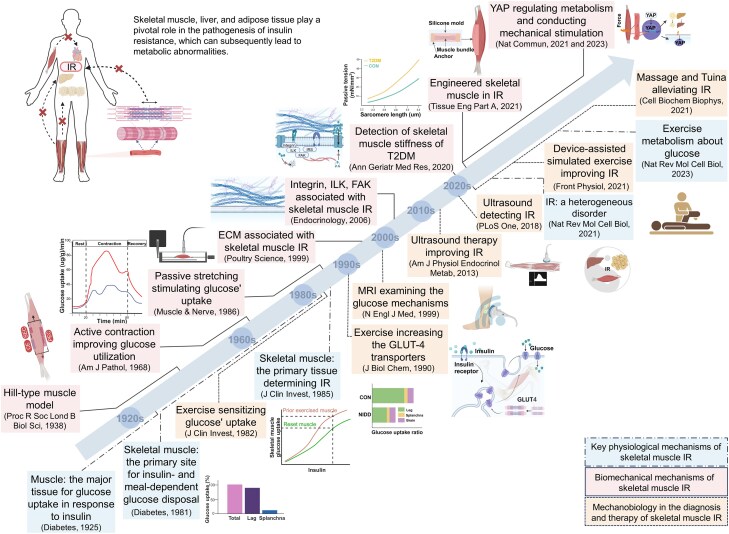
Evolution of mechanomedicine for skeletal muscle insulin resistance (IR). Key milestones in the biomechanics and mechanobiology of skeletal muscle IR and their diagnostic and therapeutic applications are summarized. Dot-dash lines denote the physiological mechanisms underlying skeletal muscle IR. Solid lines highlight crucial research in biomechanics and mechanobiology related to skeletal muscle IR. Dotted lines represent the application of mechanodiagnostic tools and mechanotherapies in managing skeletal muscle IR. Adapted and used with permission under the Creative Commons Attribution 4.0 International License (CC BY-4.0) (https://creativecommons.org/licenses/by/4.0/) (13). Adapted and used with permission from the Copyright Clearance Center, Inc. (35, 36). Created with BioRender.

The evolution of our understanding of skeletal muscle IR has been substantially enhanced by integrating the innovative emerging domain of mechanomedicine. This progression is underscored by relevant pivotal scientific achievements (Fig. 1). Skeletal muscle IR research began in 1925 with the discovery that skeletal muscles are a primary tissue through which insulin facilitates glucose uptake, highlighting their central role in metabolic control (37). Considerable advancements were made in 1981, when skeletal muscle was identified as the main site for insulin-stimulated and meal-dependent glucose disposal in humans, accounting for 85% of the total glucose metabolism (38, 39). However, skeletal muscle was not implicated as the key tissue in IR until 1985 when leg glucose uptake was found to be 45% lower in a T2DM group than in a control group (35). Glucose uptake and utilization by skeletal muscles also were found to have been promoted. In 1990, a study found that glucose transporter 4 (GLUT4) expression on muscle membranes was enhanced by insulin and physical exercise (40). Then, in 1999, a reduced rate of glycogen synthesis was identified in the skeletal muscles of patients with T2DM (41). These milestones highlight the critical role of skeletal muscle in glucose metabolism.

The exploration of biomechanics in the context of skeletal muscle IR has its roots in the important contributions of the 1930s, such as development of the Hill-type muscle model in 1938, which refers to the 3-element system: a contractile element, an elastic spring element, and a parallel elastic element. This model is characterized by force-velocity and force-length relationships, making it useful for simulating muscle behavior under various conditions. Since then, the model has been continuously refined and validated to enhance its accuracy and applicability (42, 43). Subsequent research in 1968 and 1986 revealed that both active contraction and passive stretching of skeletal muscles could improve glucose utilization and enhance uptake, respectively, underscoring the mechanobiological aspects of muscle function in glucose metabolism (36, 44). In 1982, it was discovered that exercise substantially enhanced glucose uptake by the skeletal muscle and increased its insulin sensitivity (45). In the 2010s, the general research focus expanded to the molecular level, with studies revealing the significant roles of ECM (eg, deposition and cross-linking of collagen) and its mechanosensitive proteins (eg, integrin, integrin-linked kinase [ILK], and focal adhesion kinase [FAK] in the development of skeletal muscle IR (46, 47). In the 2020s, Yes-associated protein (YAP), a mechanoreceptor protein, was identified as a key factor in regulating metabolic processes related to skeletal muscle IR and in contributing to mechanobiological signaling within muscle tissue. YAP receives upstream mechanical stimulation and induces a downstream mechanical response by entering the nucleus (34, 48). Concurrent research noted elevated stiffness in skeletal muscles affected by IR, indicating a biomechanical alteration associated with the condition (13). Subsequently, numerous studies have increasingly focused on investigating the interplay between skeletal muscle mechanobiological pathways and glucose metabolism.

Advancements in the biomechanics and mechanobiology of skeletal muscle IR have driven increased exploration of mechanodiagnosis and mechanotherapy. These developments have progressed from the use of MRI in the late 20th century to the use of ultrasound in the 2020s for detecting muscle-stiffness abnormalities (41, 49). Evolution of therapeutic interventions has led to innovative approaches, such as ultrasound-targeted microbubble destruction, simulated movement, and Tuina, enabling treatments for skeletal muscle IR (50-54). Alongside these advancements, there has been substantial progress in the mechanical understanding of skeletal muscle metabolism, molecular responses to exercise, and the factors contributing to IR (1, 55). This multidisciplinary progression from biomechanics and mechanobiology to mechanodiagnosis and mechanotherapy provides a comprehensive framework for understanding and addressing IR.

## Biomechanics of Skeletal Muscle Insulin Resistance

### Stiffness of skeletal muscle insulin resistance

**Figure 2.**
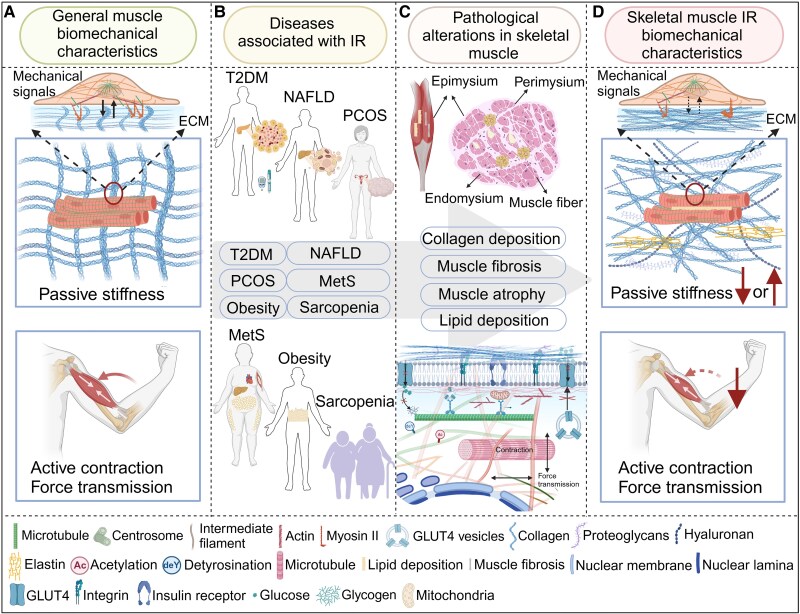
Biomechanics of skeletal muscle and its relation to skeletal muscle insulin resistance (IR). A, Biomechanical cues of skeletal muscle under normal physiological conditions, including stiffness and active contraction. B, Various diseases related to skeletal muscle IR. C, Pathological changes in skeletal muscle IR. At the tissue level, these changes include collagen deposition, fibrosis, atrophy, and lipid deposition, and at the cellular level, they involve cytoskeletal detyrosination and acetylation, branched F-actin formation, integrin inactivation, and decreased GLUT4 translocation to the plasma membrane. D, Alterations in the biomechanical cues of skeletal muscle IR, including in modified stiffness and reduced contractility. MetS, metabolic syndrome; NAFLD, nonalcoholic fatty liver disease; PCOS, polycystic ovary syndrome; T2DM, type 2 diabetes mellitus. Created with BioRender.

The relationship between skeletal muscle stiffness and IR has been extensively studied. However, results from the use of different detection methods at various biological scales, such as the in vivo organ, in vitro tissue, and cellular levels, show inconsistencies (Fig. 2A) (13, 28, 56-63). The nonuniformity of stiffness across scales can probably be attributed to distinct governing factors at different scales, which can be observed in various diseases and organizational structures (64). The multifactorial effect on stiffness emphasizes the intricacy of interpreting the mechanical properties in skeletal muscle tissues.

At the organ level, various factors (eg, adipose deposits, muscle edema, atrophy and impaired functional activity) significantly affect muscle tissue stiffness in vivo (65). Ultrasound, particularly shear wave elastography (SWE), has provided valuable insights into the mechanical properties of tissues under various pathological conditions, such as T2DM and NAFLD (2, 3, 66). Therefore, SWE has been increasingly used in clinical research to assess skeletal muscle stiffness (66). The occurrence of skeletal muscle IR is linked to various clusters of abnormal syndromes. Studies have consistently demonstrated that patients with T2DM exhibit decreased skeletal muscle stiffness (57-61), and if complications are present, these patients experience greater reductions in skeletal muscle stiffness (32, 67). Conversely, in the context of cirrhosis (contributing to IR of liver and skeletal muscle), patients exhibit greater skeletal muscle stiffness than individuals without IR (62). Although ultrasound is valuable for assessing skeletal muscle stiffness, the lack of standardized methodology can lead to variable results across different studies (68), underscoring the critical need for standardized methodologies. The changes in skeletal muscle IR biomechanics, both in the temporal scale and etiology, imply integration of mechanical and biological processes. Therefore, detection of skeletal muscle stiffness to guide treatment is reliable.

At the tissue level, the ECM is now widely recognized as the primary contributor to skeletal muscle stiffness (29, 43, 69-71). Skeletal muscle ECM consists of the epimysium, perimysium, and endomysium and is crucial for structural integrity and function since its dysregulation is linked to metabolic disorders, particularly T2DM (24). Direct comparisons of the stiffness of the gluteus maximus muscles have demonstrated an approximately twice as high increase in patients with T2DM relative to those without T2DM, which was primarily attributed to changes in the ECM, such as collagen and fatty deposition (13). Several diseases are also associated with abnormal ECM composition in skeletal muscle IR, including NAFLD (3), polycystic ovary syndrome (PCOS) (72), sarcopenia (5), obesity (73), and metabolic syndrome (74) (Fig. 2B). Understanding these relationships is crucial for developing targeted interventions that address the biomechanical aspects of muscle dysfunction in metabolic disorders.

Increased ECM stiffness in skeletal muscle IR is a multifactorial response influenced by ECM components (eg, collagen deposition, cross-linking, fatty deposition) and inflammatory factors within and around muscle fibers (75). Transforming growth factor β has a critical role in regulating fibrosis and inflammation across various tissues and induces high expression of collagen and muscle fibrosis in skeletal muscle IR (25, 72, 76-78). Chronic inflammation is another hallmark of skeletal muscle IR, characterized by elevated levels of inflammatory factors, such as interleukin-1β and components of the NLRP3 inflammasome. These inflammatory mediators further contribute to ECM deposition and fibrosis, triggering increased stiffness and worsening functional alterations in skeletal muscle IR (79, 80). Dysregulated activation of fibro-adipogenic progenitors can also differentiate into adipocytes and fibrotic cells, promoting ectopic lipid accumulation and fibrosis within the skeletal muscle IR (27, 81). Advanced glycation end products can alter the tertiary structure of collagen and enhance the cross-linking of collagen fibers as well as reduce their susceptibility to breakdown by matrix metalloproteinases (MMPs). This leads to an accumulation of collagen and increased ECM stiffness, significantly contributing to the pathophysiology of skeletal muscle IR (82-85). The role of MMPs in ECM remodeling is also crucial as they are responsible for degrading collagen and other ECM components. Reduced MMP-9 activity is directly associated with impaired collagen degradation and contributes to increased ECM stiffness and skeletal muscle IR (86, 87). These insights highlight the critical role of the ECM in modulating skeletal muscle stiffness and how metabolic alterations associated with IR can lead to substantial changes in muscle biomechanics (13) (Fig. 2C). The various study findings underscore the importance of understanding the complex interplay between mechanical and biochemical factors in skeletal muscle IR, which would then support development of more effective and targeted therapeutic strategies.

Abnormal skeletal muscle cellular stiffness is also directly related to IR (56). The cytoskeleton, comprising actin filaments, intermediate filaments, and microtubules, has a crucial role in determining cellular stiffness (56). Atomic force microscopy tests have revealed significantly elevated stiffness in insulin-resistant skeletal muscle cells, which contain more branched F-actin with a greater load-bearing capacity than linear filaments (88). The stiffened cortical actin structure in insulin-resistant cells may function as a significant physical barrier for GLUT4 storage vesicles (GSVs) to integrate into the plasma membranes (PMs), indicating a defective step in IR (56, 88). In another study, the passive stiffness of the soleus muscle was elevated in desmin knockout mouse (89). Disease-induced changes in microtubules (eg, detyrosination and acetylation) alter cytoskeletal stiffness. Insulin and IR induce modifications that promote polymerization, stabilize microtubules, and increase their density and curvature, leading to increased cellular stiffness (28, 90-94). Although there is no direct evidence, IR cells may alter skeletal muscle stiffness through changes in microtubules (92). The cytoskeleton generally serves as a central hub for cellular stiffness regulation and GSV transport and is essentially a shared glucose metabolism and biomechanical pathway in skeletal muscle IR. This convergence offers a unique avenue for improving insulin sensitivity.

### Active contraction of skeletal muscle insulin resistance

Muscle fibers are the essential structural and functional units that facilitate movement through the process of myofilament sliding, which occurs almost simultaneously among numerous fibers within a motor unit to produce muscle contraction. In the context of IR, skeletal muscle fibers are encased in an altered ECM, significantly affecting their structure and function. This altered ECM composition can influence the mechanical and biochemical microenvironment of the muscle cells, leading to impaired glucose uptake and metabolism. For example, IR induces a reduction in the muscle contractile force, as observed in both in vitro and in vivo studies (Fig. 2D). This reduction is a direct consequence of the compromised structural and functional integrity of the muscle fibers, as induced by the pathological changes in the ECM and fibrofatty degeneration (27, 95-97).

The generation and transmission of force in the skeletal muscle are critical for its proper functioning, and both processes are notably affected by IR (89, 98). The active force generated within a muscle fiber originates from individual actin-myosin cross-bridges, which are then transmitted both longitudinally and laterally within the fiber (99). Longitudinal-force transmission runs the length of the muscle fiber between the z-discs and to the tendon via the myotendinous junction to initiate movement. Lateral-force transmission, which disperses the force across the width of the muscle fiber, follows a path from the z-discs through the cytoskeleton and intermediate filament desmin, extending out to the ECM via the costamere (12, 89). Notably, approximately 80% of the force generated by the muscle fiber reportedly is transmitted laterally (12). The transmission of this force is not confined to the muscle fiber's internal structures but extends to the cell membrane through integrins and dystrophin (100-102), facilitating force transmission to the cell membrane, which is crucial for promoting muscle contraction. However, in the skeletal muscle affected by IR, the normal architecture and functionality of these components are compromised. Accumulation and disorganization of collagen fibers within the ECM in the presence of IR, combined with the aberrant expression of integrins and dystrophin, can impede efficient lateral-force transmission, hindering skeletal muscle contractile efficacy (89, 98).

## Mechanobiology of Skeletal Muscle Insulin Resistance

### Mechanobiology of stiffness in skeletal muscle insulin resistance

**Figure 3.**
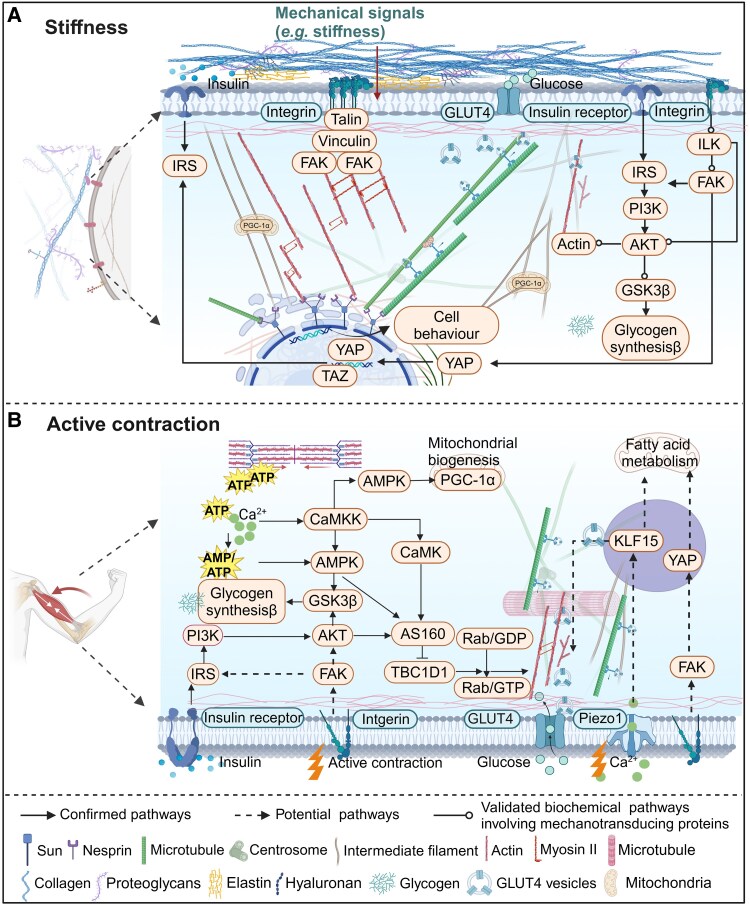
Mechanobiology of skeletal muscle insulin resistance (IR). The mechanobiology observed in skeletal muscle IR and potential crosstalk between mechanobiological and physiological pathways. A, Integrins in ECM undergo mechanical stimulation, leading to their engagement in multitransmembrane complexes (eg, focal adhesions [FAs]). FAs transmit mechanical signals through the cytoskeleton to the nucleus, ultimately modulating nuclear transcription factors and other factors, thereby influencing cellular behavior and metabolism. The typical mechanical pathway (integrins/ILK/FAK/YAP) and the mechanoreceptor cytoskeleton intersect with the insulin pathway and mitochondrial function in skeletal muscle, representing a potential target for mechanical signals to impact insulin sensitivity in skeletal muscle. B, active skeletal muscle contraction may stimulate integrins and their downstream mechanical pathways, as well as activate Piezo1 to facilitate Ca^2+^ influx. This process preserves the contractility of skeletal muscle, enhances glucose uptake, and ameliorates IR. Moreover, the convergence between mechanical signaling and classical exercise pathways might synergistically improves glucose metabolism, presenting a potential mechanism for enhancing skeletal muscle IR. AKT, serine/threonine kinase; AMPK, AMP-activated protein kinase; AS160, Akt substrate of 160 kDa; CaMK, Ca^2+^/calmodulin-dependent protein kinase; CaMKK, CaM-dependent protein kinase kinase; ECM, extracellular matrix; FAK, focal adhesion kinase; GLUT4, glucose transporter 4; GSK3β, glycogen synthase kinase-3 β; ILK, integrin-linked kinase; IRS, insulin receptor substrate; KLF15, Kruppel-like factor 15; PI3K, phosphatidylinositol 3-kinase; Piezo1, piezo-type mechanosensitive ion channel component 1; PGC-1α, Peroxisome proliferator-activated receptor γ coactivator 1-α; TAZ, transcriptional coactivator with PDZ-binding motif; TBC1D1, TBC1 domain family member 1; YAP, Yes-associated protein. Solid lines with arrow depict confirmed pathways, solid lines with circle depict validated biochemical pathways involving mechanosensitive proteins, while dashed lines represent potential pathways. Created with BioRender.

The abnormal stiffness of the ECM in IR has a profound effect on skeletal muscle cells (103). Mechanotransduction processes enable skeletal muscle cells to mechanosensitively couple ECM stiffness with the actin cytoskeleton through multitransmembrane complexes, such as cell-ECM adhesions. These complexes consist of integrins, receptor tyrosine kinases, and other proteins, facilitating mechanical signals transmission from the ECM to the cytoskeleton (104, 105). As a type of cell-ECM adhesion, focal adhesions (FAs) have a central role in sensing biomechanical cues (eg, ECM stiffness), and changes in FAs subsequently trigger cellular responses, including cytoskeleton reorganization and cytoplasmic signaling cascades (Fig. 3A and Table 1) (105). ECM stiffness regulates mitochondrial morphology, promoting fusion and suppressing fission (123). The integrin-cytoskeleton connection transmits forces to the mitochondria, and vimentin and desmin are involved in regulating mitochondrial performance (12, 92, 103). Therefore, changes in mitochondrial morphology and dynamics substantially affect insulin responsiveness in skeletal muscle IR (124). Mice with IR have softer skeletal muscle, but a stiffer matrix enhances cytoskeletal transport, promoting mitochondrial dispersion and GLUT4 translocation and improving glucose uptake relative to that of softer matrices (28). High ECM stiffness exacerbates IR progression by inhibiting GSVs and mitochondria transport via the cytoskeleton and multitransmembrane proteins within the ECM. In skeletal muscle IR, altered stiffness translates mechanical cues in their microenvironment into biochemical signals through FAs and the cytoskeleton, thereby affecting metabolic mechanisms.

**Table 1.** Mechanistic pathways in the mechanobiology of skeletal muscle insulin resistance

Category	Pathway	Description	References
Confirmed pathways	IRR/IRS/YAP/TAZ	TAZ couples Hippo/Wnt signaling and insulin sensitivity through IRS1 expression	(106)
	IRR/IRS/PI3 K/AKT/GSK3β	The insulin pathway is involved in glycogen synthesis	(1, 107)
	IRR/IRS/PI3 K/AKT/AS160/TBC1D1/(Rab/GDP)/F-actin/GLUT4	The insulin pathway is involved in glucose uptake	(1, 107)
	Ca^2+^/CaMKK/CaMK/AS160	Ca^2+^ and AMPK both mediate stimulation of glucose transport by muscle contractions	(108-114)
	Integrin/Talin/Vinculin/FAK/F-actin	Integrin and other proteins assemble protein complexes at adhesion sites to transmit mechanical signals	(11, 12, 102)
	Integrin/FAK/IRS	Investigate whether integrin, when stimulated by mechanical signals, can participate in insulin signaling through promotion of IRS phosphorylation in skeletal muscle	(11, 12, 115)
Potential pathways	Integrin/FAK/F-actin/GLUT4	Investigate if integrin stimulated by mechanical signals can facilitate translocation of GLUT4 to the PM through F-actin, thereby enhancing glucose uptake capacity in skeletal muscle	(11, 12, 116)
	Integrin/FAK/YAP	Integrins detect mechanical stimuli and transmit mechanical signals to the nucleus, leading to nuclear translocation of YAP and subsequent alterations in transcriptome	(11, 12, 117)
	Piezo1/KLF15/F-actin/GLUT4	Explores Piezo1-mediated mechanosignaling promoting GLUT4 translocation via F-actin remodeling	(118-120)
	Piezo1/KLF15/Fatty acid metabolism	Examines role of Piezo1 and KLF15 in regulating fatty acid metabolism	(118-120)
	YAP/fatty acid oxidation	YAP regulates skeletal muscle fatty acid oxidation and adiposity in metabolic disease	(48)
Validated biochemical pathways involving mechanotransducing proteins	Integrin/FAK/PI3K/F-actin/GLUT4	FAK contributes to insulin-induced actin reorganization into a mesh harboring GLUT4 in insulin-resistant skeletal muscle cells.	(116)
	FAK/IRS1/AKT	Phosphorylation and expression of IRS-1 and Akt-Ser473-phosphorylation decreased in FAK-silenced mice	(115)
	ILK/GLUT4	Peripheral insulin resistance in ILK-depleted mice by reduction of GLUT4 expression	(121, 122)

Abbreviations: AKT, serine/threonine kinase; AMPK, AMP-activated protein kinase; AS160, Akt substrate of 160 kDa; CaMK, Ca2+/calmodulin-dependent protein kinase; CaMKK, CaM-dependent protein kinase kinase; FAK, focal adhesion kinase; GLUT4, glucose transporter 4; GSK3β, glycogen synthase kinase-3 β; ILK, integrin-linked kinase; IRR, insulin receptor; IRS, insulin receptor substrate; KLF15, Kruppel-like factor 15; PI3K, phosphatidylinositol 3-kinase; Piezo1, piezo-type mechanosensitive ion channel component 1; TAZ, WW-domain–containing transcription regulator 1; TBC1D1, TBC1 domain family member 1; YAP, Yes-associated protein.

The mechanobiology of skeletal muscle IR encompasses significant nuclear aberrations. The linker of nucleoskeleton and cytoskeleton (LINC) complexes have a crucial role in facilitating transmission of biomechanical cues to the nucleus (125), which in turn influences chromosome organization and gene expression (126, 127). Expression of nesprin, a structural component of the LINC complex, is significantly lower in skeletal muscle IR than in healthy skeletal muscle. This reduction affects cellular differentiation processes, which are critical for maintaining muscle function and responding to metabolic demands (125, 126). The nuclear architecture, particularly the levels and assembly of A-type lamins, is a key determinant of the nuclear stiffness and viscoelastic properties, influencing the nucleus' ability to respond to mechanical signals (127, 128). Although genome-wide association studies have suggested a link between nuclear lamins and the pathophysiology of T2DM, there are few detailed studies in this area (129). Additionally, increased lamin A/C in macrophages promotes active inflammation, contributing to the development of IR (130), and postexercise-enhanced lamin A expression in individuals over 75 years of age has been found to be associated with increased nuclear stiffness (131). These observations indicate that the nuclear mechanical microenvironment is altered in skeletal muscle IR. This dysfunctional mechanotransduction could affect both the biomechanical cues of the skeletal muscle and the nuclear signaling pathways that govern cellular behavior and metabolism.

Future research in mechanobiology could provide new insights into the pathogenesis of IR. Understanding these mechanisms could lead to novel therapeutic avenues, targeting the mechanical and structural components of muscle cells to improve insulin sensitivity and treat the metabolic dysfunction associated with IR.

### Mechanobiology of active contraction in skeletal muscle insulin resistance

The process of skeletal muscle active contraction involves the coordinated movement of proteins, force transmission, and glucose uptake regulation (Fig. 3B and Table 1) (108). Muscle contraction is generated by myosin and actin cross-bridges, with Ca^2+^ dynamics and adenosine triphosphate (ATP) hydrolysis having key roles. The AMP-activated protein kinase (AMPK)-related and calcium-mediated signaling pathways are crucial in skeletal muscle contraction and are integral to the mechanism by which muscle contraction facilitates GLUT4 translocation to the PM, thereby enhancing glucose uptake (108-111, 132). Downstream proteins, such as TBC1D4 (AS160)/TBC1D1 and Rac1/PAK2 that are associated with cytoskeletal actin remodeling, are also critical for GLUT4 translocation to the PM (111). The relationship between muscle contractility and glucose transport is direct, with increased contractile activity enhancing glucose uptake across various mammalian muscle fiber types (95, 108, 110, 112-114, 133, 134). In addition, impaired Ca^2+^ handling by calcium-channel proteins on the muscle-cell membrane is associated with reduced contractility in skeletal muscle during IR. The reduced contractile activity of skeletal muscle IR and atrophy is correlated with abnormalities in calcium handling, such as increased resting [Ca^2+^]_i_ (97) and induced peak Ca^2+^ value (135-139). The external calcium influx is crucial for replenishing intracellular stores, which ensures continued muscle fiber excitability and contractility (136). The increase in calcium during skeletal muscle contraction also activates several enzymes and pathways involved in insulin sensitivity. The impaired contractile function of skeletal muscle IR is associated with alterations in glucose metabolism, highlighting the importance of mechanical factors in maintaining biological health.

### Mechanosensitive proteins in the extracellular matrix of skeletal muscle insulin resistance

Cell-ECM adhesions are crucial for transducing mechanical cues from the ECM into the cell, thereby influencing cellular behavior. Activation of integrin initiates a cascade of interactions, leading to protein complex assembly at the adhesion sites. These complexes consisting of approximately 60 different proteins, including integrins, ILK, and FAK, are essential to physiological processes in skeletal muscle (11, 12, 104). The mechanosensitive proteins have a substantial role in the cellular mechanisms governing skeletal muscle glucose metabolism, atrophy, and various other physiological and pathological phenomena.

### Mechanosensitive proteins integrins

Skeletal muscle IR shows diminished expression or dysfunction of mechanosensitive proteins in ECM (2). Integrins are vital transmembrane receptors that facilitate cell adhesion by bridging the ECM to the cytoskeleton and are instrumental in the bidirectional transduction of biochemical and mechanical signals between cells and their surrounding environment (11). The structure and mechanoconduction function of integrins have been comprehensively reviewed (11, 104). Notably, the deficiency in muscle-specific integrin β1 (ITGB1) in mice has been associated with significant reductions of 44% in insulin-stimulated glucose infusion and of 48% in glucose clearance rates. These findings highlight the crucial role of ITGB1 in maintaining glucose homeostasis. Similarly, mice with dysfunctional ITGB2 also exhibited skeletal muscle IR (140). Conversely, a reduction in ITGA2 expression in IR mice correlated with improved insulin sensitivity, suggesting a potential modulatory role of ITGA2 in insulin signaling (87). Furthermore, early IR development may involve integrin modulation of insulin action. Healthy males with lower insulin sensitivity have elevated expressions of ITGA5-7, indicating a correlation between specific integrin expressions and insulin sensitivity in the skeletal muscle (141). ITGB1 deficiency impairs insulin-stimulated insulin receptor substrate (IRS) phosphorylation and downstream phosphatidylinositol 3-kinase (PI3K) and protein kinase B activation (142). The interaction between ITGAVB5 and the insulin receptor has been shown to disrupt skeletal muscle insulin receptor signaling (143). These results indicate a possible correlation between specific integrin expressions and insulin sensitivity levels in skeletal muscle.

### Mechanosensitive protein integrin-linked kinase

ILK is a crucial intracellular scaffold protein that functions downstream of integrin signaling by exerting a considerable mechanotransduction effect in the IR pathways of skeletal muscle (11, 144). Skeletal muscle ILK expression has been found to decrease early and progressively in IR mice (121), whereas ILK-inhibited mice have shown reduced peripheral insulin sensitivity and glucose uptake (122). Reduction in ILK leads to decreased insulin sensitivity and glucose uptake, establishing a direct correlation between ILK function and glucose metabolism (121, 122, 145). Bioinformatics analyses also have expanded our understanding of ILK's role by showing that the ILK pathway, along with glycolysis and gluconeogenesis, is associated with biological processes linked to extracellular vesicles–associated microRNAs that undergo changes in gestational diabetes mellitus. This association indicates that ILK is involved both in the direct regulation of glucose metabolism within muscle cells and possibly in broader metabolic and signaling networks, influencing the pathogenesis of IR and associated metabolic disorders (146).

### Mechanosensitive protein focal adhesion kinase

The tyrosine kinase, FAK, is situated downstream of integrin and has a crucial role in intracellular signaling, cytoskeletal stabilization, and FA turnover (2). Cells and animals that are IR have shown a notable decrease in tyrosine phosphorylation of FAK, and in vivo and in vitro inhibition of FAK was shown to cause IR (115, 116, 147). Conversely, experiments in which enhanced FAK activity was shown to improve IR in skeletal muscle highlight the potential of targeting FAK to ameliorate IR (115, 116, 147). FAK improves insulin sensitivity by facilitating actin and cytoskeletal reorganization and interacting with downstream signaling molecules, such as IRS-1, PI3K, PKC, and GSK3β, which are crucial for GLUT4 translocation and glucose uptake (47, 115, 116, 147). The influence of the ITGB1-FAK pathway on intracellular cytoskeletal organization also extends to mitochondrial function, linking biomechanical signaling to cellular energy metabolism and insulin sensitivity (148). The dynamic interplay between FAK and insulin signaling receptors indicates a novel and promising avenue for treating skeletal muscle IR.

### Mechanosensitive proteins Piezo-type mechanosensitive ion channel component 1

Piezo-type mechanosensitive ion channel component 1 (Piezo1) has emerged as a mechanically activated Ca^2+^ channel (118, 149-152) and interacts with the intracellular cytoskeleton to transmit mechanical signals (153). On mechanical stimulation (eg, exercise), the activation of Piezo1 leads to an increase in [Ca^2+^]i (150), potentially improving peak Ca^2+^ values. As discussed earlier, external calcium influx is essential for maintaining muscle fiber excitability and contractility. Exercise, by enhancing Piezo1 activation, may promote better Ca^2+^ regulation and, consequently, improve muscle function and contractility in the context of IR. Piezo1 is also crucial in regulating glucose uptake. F-actin serves as a mechanical conduit for Piezo1 channels to transmit mechanical signals and facilitates GLUT4 trafficking to the PM. These functions suggest that Piezo1 may be involved in skeletal muscle IR through F-actin dysfunction (93, 107, 150, 153), as illustrated in Fig. 3B and Table 1. Since Kruppel-like factor 15 (KLF15) and interleukin-6 (IL-6) are recognized as key regulators of metabolic processes in muscle tissue, particularly in relation to IR, emerging research suggests a novel Piezo1/KLF15/IL-6 signaling axis that may influence muscle metabolism, with implications for GLUT4 translocation, and offers insights into new molecular pathways implicated in skeletal muscle IR (118-120). The activation of Piezo1 by skeletal muscle contraction regulates impaired contractility and glucose uptake in skeletal muscle IR through calcium influx and its interaction with the cytoskeleton. Mechanosensitive channels detect mechanical stimuli and transduce them into downstream biochemical pathways, offering novel avenues for improving IR.

### Mechanosensitive proteins Yes-associated protein

Cell-ECM adhesions transmit biomechanical cues and ultimately modulate transcription factors, influencing cellular behavior and metabolism. The nuclear translocation of YAP/TAZ further exemplifies how mechanotransduction affects muscle function (117). In particular, YAP has been shown to regulate fatty acid oxidation and lipotoxicity; therefore, YAP modulation potentially can provide therapeutic treatment approaches to IR in obese mice (48). Similarly, the interactions of transcriptional coactivator with PDZ-binding motif (TAZ) and signaling molecules influence IRS1, serine/threonine kinase (AKT), and GLUT4 (106). Given the documented role of YAP in myocardial fibrosis through a mechanobiological feedback loop involving ITGB1 and Piezo1, it is plausible to explore similar regulatory mechanisms in skeletal muscle (29). Given that cell membranes and cells contain a diverse array of mechanosensitive proteins, which have crucial roles in mechanotransduction, this is an area requiring further research.

Skeletal muscle IR not only results from metabolic dysfunctions but also induces important changes in the mechanical microenvironment and behavior of mechanosensitive proteins. These mechanosensitive proteins, which are crucial for sensing and responding to biomechanical cues, are integral to the cellular processes that govern muscle function and metabolism. The aberrant mechanical microenvironment in skeletal muscle with IR can alter the function of these mechanosensitive proteins, potentially disrupting their normal role in transducing mechanical signals into biochemical responses, which could contribute to the pathogenesis of skeletal muscle IR. This possibility would require a bidirectional relationship in which IR affects the biomechanical cues of the muscle, which in turn influences the functionality of the mechanobiological pathways, creating a feedback loop that exacerbates IR. Future research could focus on elucidating the precise mechanisms by which mechanosensitive proteins sense and relay mechanical stimuli under IR conditions.

## Mechanodiagnosis and Mechanotherapy for Treating Skeletal Muscle Insulin Resistance

### Mechanodiagnosis of skeletal muscle insulin resistance

**Figure 4.**
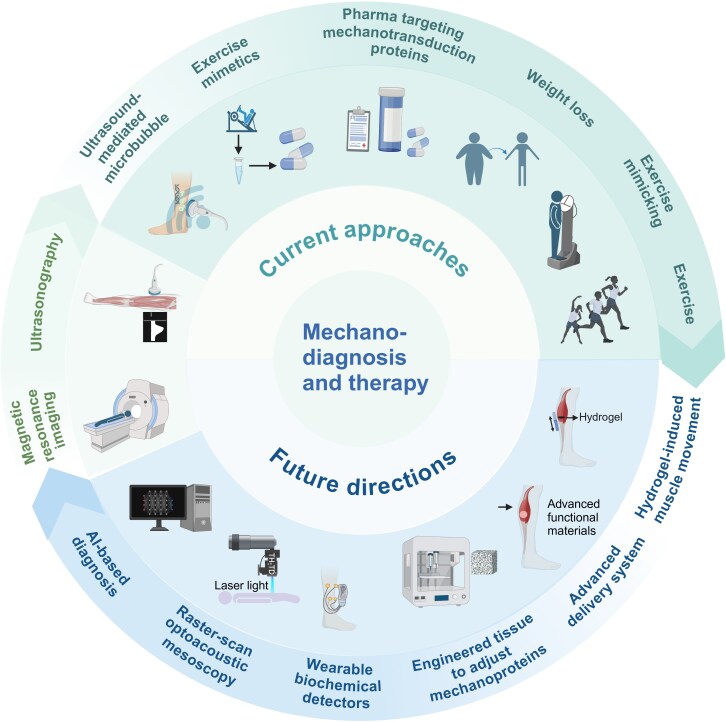
Current and future mechanodiagnosis and mechanotherapy of skeletal muscle insulin resistance (IR). Current mechanotherapies for skeletal muscle IR include exercise, exercise mimicking, medications targeting mechanosensitive proteins, exercise mimetics, and ultrasound-mediated microbubble therapies, as depicted in the upper section. Mechanodiagnostic modalities, such as ultrasonography and magnetic resonance imaging, are illustrated on the left. Emerging prospective approaches—including hydrogel-based delivery systems, optoacoustic mesoscopy, wearable biochemical detectors, engineered tissue-based diagnosis, and artificial intelligence–guided mechanotherapies and diagnoses—are presented in the lower section. Created with BioRender.

Due to technical limitations, the utilization of mechanical changes for diagnostics is not currently widespread. Common methods used for quantitative detection of stiffness include atomic force microscopy, which is limited to invasive ex vivo measurements from a freshly isolated biopsy sample, and provides information only about specific regions within the selected biopsy (154). However, MRI and SWE have been increasingly validated in recent years through clinical studies for the detection/characterization of tissue biomechanical properties (57, 154). The validity of SWE and MRI for evaluating skeletal muscle stiffness, especially in the context of IR, also has been clinically investigated. These modalities offer noninvasive means to quantify the mechanical properties of tissues, providing valuable insights into the biomechanical alterations associated with metabolic conditions. Ultrasound SWE has demonstrated its utility in detecting variations in muscle stiffness among individuals, showing considerable differences between those with IR and healthy controls. These findings underscore the potential of SWE as a reliable tool for identifying biomechanical changes in skeletal muscle associated with IR. Similarly, MRI has been performed to assess muscle stiffness and provides data complementary to ultrasound SWE data. Although MRI offers detailed tissue characterization and is particularly useful for assessing deeper muscle structures, it faces challenges, such as variability in measurement protocols and interpretation of results (Fig. 4) (155-158). Despite the promise shown by these imaging techniques in individual studies, the field faces challenges in standardization, particularly when comparing results across different research efforts. The lack of uniform protocols and measurement parameters can lead to variability in the findings, complicating the task of drawing broad conclusions.

To enhance the reliability and comparability of muscle stiffness assessments, standardized protocols for the use of ultrasound SWE and MRI are needed. Establishing consistent methodologies would enable more accurate cross-sectional comparisons and longitudinal tracking of changes in muscle stiffness across diverse populations and conditions. Although ultrasound SWE and MRI hold significant potential for assessing stiffness in IR skeletal muscle, advancing the field requires a concerted standardization effort and methodological rigor. Such advancements would improve the clinical and research utility of these imaging techniques, offering deeper insights into the biomechanical aspects of muscle function and disease.

### Mechanotherapy to treat skeletal muscle insulin resistance

Considering the mechanobiological factors influencing skeletal muscle IR, potential strategies to enhance insulin sensitivity include reducing collagen deposition and cross-linking in the ECM, enhancing the mechanotransduction processes at cell-ECM adhesions, and bolstering the interplay between muscle contraction and glucose metabolism (see Fig. 4). This section explores mechanotherapeutic strategies to improve skeletal muscle IR from the tissue level (various exercise modalities) to the cellular and molecular levels (medications targeting ECM stiffness and mechanosensitive proteins and mechanical therapy).

The utilization of diverse exercise modalities, including active exercise (eg, aerobic and resistance exercises), passive stretching, and instrument-assisted exercise simulation, can effectively improve skeletal muscle IR through various mechanisms. Active exercise promotes rapid turnover of collagen, enhances degradation of structural proteins, and helps to significantly reduce lipid accumulation, fibrosis, and inflammation (, 89, 89, 159-165). Active exercise also has been shown to correct impairments in muscle contractile force and calcium regulation in a T2DM animal model (97). Passive stretching of skeletal muscle tissues or cells in vitro or in vivo can effectively simulate localized exercise-induced benefits on IR (166). Mechanical stretching in vivo has been shown to enhance glucose uptake in skeletal muscle IR by approximately 80% (167, 168). The mechanism involves multiple pathways (eg, activation of Rac1) and remains unaffected by how exercise influences skeletal muscle vasculature to improve IR (112, 166, 169-173). Instrument-assisted exercise mimicking can effectively alleviate muscle IR (174). The focus of jogging devices and enhanced external counterpulsation has primarily been on their vascular effects for metabolic benefits; however, it must be acknowledged that these modalities also elicit passive skeletal muscle movements (175, 176). “Relaxing-Vibration training” using a vibration plate to induce stretching improved the levels of metabolic glucose regulators associated with myokines, muscle stiffness, and glycemic management (174). Different types of exercise use various mechanical stimulation methods to enhance skeletal muscle IR. Exploring safer and more effective mechanical stimulation techniques is a promising avenue for diagnosis and treatment.

Exercise, when combined with various approaches, can significantly reduce body weight. Weight loss has been widely shown to improve metabolism, such as glycemic control; however, it may also lead to a reduction in muscle mass, which appears to conflict with efforts to improve skeletal muscle IR (177-179). Several strategies, including dietary interventions, bariatric surgery, pharmacotherapy, and exercise, have been proposed to manage weight (180). Caloric restriction, induced by methods, such as dietary interventions and bariatric surgery, significantly reduces skeletal muscle and visceral fat deposition. In addition, it decreases systemic inflammation and lowers circulating proinflammatory cytokines (eg, tumor necrosis factor-α, IL-6). These changes not only improve skeletal muscle insulin sensitivity (181-183) but also modify ECM components (eg, reducing fibrosis) (184) and may improve cytoskeletal structure (185), potentially regulating the skeletal muscle stiffness associated with IR and enhancing the skeletal muscle contractility impaired by IR (186). The combination of bariatric surgery with postoperative exercise has an additive effect on promoting ECM remodeling (eg, reducing collagen deposition), thereby regulating skeletal muscle stiffness (76). Pharmacotherapy for weight loss, primarily through glucagon-like peptide-1 (GLP-1) receptor agonists, markedly promotes insulin sensitivity and reduces muscle fat infiltration, probably contributing to enhanced skeletal muscle contractility (187, 188). Despite some contradictory findings regarding whether GLP-1 can improve muscle mass (189), other studies suggest that muscle mass and strength do not always change proportionally due to factors, such as obesity and metabolic abnormalities (190). Therefore, it is crucial to determine whether a reduction in skeletal muscle mass can coexist with improvements in skeletal muscle strength and metabolic capacity during weight loss. Additionally, integrating multimodal strategies to optimize weight loss while preserving muscle function and metabolic health is essential.

Significant advancements have also been made in targeted therapy at the molecular level through medications targeting ECM stiffness and mechanosensitive proteins and mechanical therapy. Interventions, such as hyaluronidase PEGPH20 (75), sildenafil (87), metformin, and telmisartan (191), can significantly reduce the deposition of collagen types I and III in the ECM of skeletal muscle IR, thus reducing ECM stiffness and ultimately improving metabolism. Applying an RGD synthetic peptide, which binds to integrin, in IR mice improved glucose clearance and insulin sensitivity, thus demonstrating the potential of targeting mechanosensitive proteins, such as integrin, for metabolic regulation (192). Moreover, exercise-mimicking drugs offer a promising approach to simulating the molecular changes in skeletal muscle induced by physical exercise (193, 194). Drugs, such as 5-aminoimidazole-4-carboxamide ribonucleotide, metformin (both targeting AMPK), and GW501516, which targets peroxisome proliferator-activated receptor (PPAR-δ), have been studied for enhancing muscle contractility, reducing fibrosis, and improving insulin sensitivity (193-202). As a form of mechanical therapy, ultrasound-targeted microbubble destruction has been used to treat skeletal muscle IR (50, 51). When microbubbles encapsulating the adiponectin gene are targeted and disrupted by ultrasound near the skeletal muscle, the therapeutic genes are released, leading to metabolic improvements. Although these findings are promising, it is important to acknowledge that the mechanobiology underlying skeletal muscle IR is not fully understood.

The interactions between biomechanical cues and mechanobiological responses in the context of IR require further elucidation. Delving deeper into the mechanobiological aspects of skeletal muscle could reveal new dimensions of IR and lead to innovative therapeutic approaches. This exploration is crucial for developing more targeted and effective treatments for IR, aligning with the growing recognition of mechanobiology's role in health and disease.

## Conclusions and Future Perspectives

Despite numerous strategies to prevent IR, including lifestyle modifications, physical activity, and pharmacotherapy, the increasing prevalence of IR and related metabolic disorders suggests a gap in our understanding of its pathophysiology (1, 203). This gap indicates the existence of unidentified pathological mechanisms and highlights the need for innovative IR treatment approaches. Although some studies have used robust biomaterials and tissue engineering to investigate skeletal muscle biomechanics, the comprehensive relationship between mechanomedicine and IR remains underexplored (204). Further research could focus on detecting biomechanical cues, such as stretching and contractility, and investigating how skeletal muscle cells integrate mechanobiological processes in response to biomechanics with glucose regulation and insulin-signaling pathways. These investigations potentially can lead to novel mechanotherapeutic interventions, including targeted exercise regimens, biomechanically inspired medical devices, or pharmacological agents that mimic the beneficial effects of mechanical stimuli on muscle tissue (see Fig. 4).

### Future perspectives on the mechanodiagnosis of skeletal muscle insulin resistance

Optoacoustic mesoscopy (OPAM) transcends the limitations of traditional optical microscopy by offering deep-tissue penetration (205). OPAM has shown promise in musculoskeletal research, particularly in investigating diseases, such as muscle atrophy and the accumulation of collagen in skeletal muscles (206-210). These capabilities indicate OPAM's utility in studying changes in muscle structure and composition that are relevant to metabolic disorders, including IR (211). Artificial intelligence (AI) also has considerable potential for diagnosing and predicting skeletal muscle IR by analyzing images from SWE, MRI, computed tomography, OPAM, and immunohistochemistry. Moreover, AI can be used with implanted advanced materials, such as hydrogels and chips to dynamically monitor the mechanical properties of skeletal muscle IR in real time, enabling accurate and personalized diagnosis and treatment plans based on this information (212). Given the potential of fundus photography combined with AI in diagnosing various tissue diseases, it is also worth assessing whether this technique can be used to diagnose skeletal muscle IR (213). The use of wearable devices for noninvasive detection of biomechanical cues (eg, stiffness and contractility) in skeletal muscles with IR is also a prospective avenue for future exploration (214, 215).

### Future perspectives on mechanotherapy for skeletal muscle insulin resistance

Hydrogel-based in vivo systems could serve as powerful tools for modulating skeletal muscle biomechanical factors and function, offering a novel mechanotherapeutic strategy for improving skeletal muscle IR. Implantable hydrogel has been shown to facilitate skeletal muscle stretching and generate muscle-contraction–mimicking stimulation with programmed strength and frequency in vivo (34, 216). These systems offer a unique approach to directly modulate skeletal muscle biomechanics. The development of hydrogel systems with adjustable stiffness has enabled the creation of adaptive anisotropic and auxetic patches that mimic the mechanical properties of healthy, dynamic skeletal muscle, aiming to enhance insulin sensitivity (217). These systems also could be developed as hydrogel dressings with embedded controlled-release drug particles for alleviating fibrosis and adipose accumulation, thereby regulating stiffness (218). Wireless magneto-active soft robots can exert mechanical forces, such as compression and stretching, on tissues, which induces skeletal muscle deformation to simulate exercise. This represents an innovative form of mechanotherapy (219). Tissue engineering can be strategically used to selectively augment receptor expression, thereby enhancing cellular functionality (8). Transplanting engineered skeletal muscle that has overexpressed mechanosensitive receptors into IR individuals may improve exercise sensitivity or augment the mechanotransduction pathway, thereby improving insulin responsiveness. This approach has shown considerable promise for addressing IR.

Further investigations are required to enable in-depth analysis of various biomechanical cues, such as viscoelasticity, of skeletal muscle under different states of insulin sensitivity. Additionally, a more detailed and accurate understanding of mechanobiology, including the sensing, transmission, and response to biomechanical cues, is necessary. These findings can support proposals for innovative and reliable diagnostic and treatment strategies that can be translated into clinical applications. The field of mechanomedicine presents a novel framework for understanding, diagnosing, and treating skeletal muscle IR.

## Abbreviations

AI: artificial intelligence
AKT: serine/threonine kinase
AMPK: AMP-activated protein kinase
AS160: Akt substrate of 160 kDa
[Ca^2+^]_I_: intracellular calcium concentration
CaMK: Ca^2+^/calmodulin-dependent protein kinase
CaMKK: Ca^2+^/calmodulin-dependent protein kinase kinase
ECM: extracellular matrix
FAs: focal adhesions
FAK: focal adhesion kinase
GLP-1: glucagon-like peptide-1
GLUT4: glucose transporter 4
GSK3β: glycogen synthase kinase-3 β
GSVs: GLUT4 storage vesicles
IL: interleukin
ILK: integrin-linked kinase
IR: insulin resistance
IRS: insulin receptor substrate
ITGB1: integrin β 1
KLF15: Kruppel-like factor 15
LINC: linker of nucleoskeleton and cytoskeleton
MMPs: matrix metalloproteinases
MRI: magnetic resonance imaging
NAFLD: nonalcoholic fatty liver disease
OPAM: optoacoustic mesoscopy
PCOS: polycystic ovary syndrome
PGC-1α: peroxisome proliferator-activated receptor γ coactivator 1-α
PI3K: phosphatidylinositol 3-kinase
Piezo1: piezo-type mechanosensitive ion channel component 1
PM: plasma membrane
SWE: shear wave elastography
T2DM: type 2 diabetes mellitus
TAZ: transcriptional coactivator with PDZ-binding motif
TBC1D1: TBC1 domain family member 1
YAP: Yes-associated protein
